# Analysis and prediction of atmospheric ozone concentrations using machine learning

**DOI:** 10.3389/fdata.2024.1469809

**Published:** 2025-01-15

**Authors:** Stephan Räss, Markus C. Leuenberger

**Affiliations:** ^1^Climate and Environmental Physics, Physics Institute, University of Bern, Bern, Switzerland; ^2^Oeschger Centre for Climate Change Research, University of Bern, Bern, Switzerland

**Keywords:** atmospheric ozone, Air Pollution Monitoring, data analysis, machine learning, artificial neural networks, multilayer perceptron, Keras

## Abstract

Atmospheric ozone chemistry involves various substances and reactions, which makes it a complex system. We analyzed data recorded by Switzerland's National Air Pollution Monitoring Network (NABEL) to showcase the capabilities of machine learning (ML) for the prediction of ozone concentrations (daily averages) and to document a general approach that can be followed by anyone facing similar problems. We evaluated various artificial neural networks and compared them to linear as well as non-linear models deduced with ML. The main analyses and the training of the models were performed on atmospheric air data recorded from 2016 to 2023 at the NABEL station Lugano-Università in Lugano, TI, Switzerland. As a first step, we used techniques like best subset selection to determine the measurement parameters that might be relevant for the prediction of ozone concentrations; in general, the parameters identified by these methods agree with atmospheric ozone chemistry. Based on these results, we constructed various models and used them to predict ozone concentrations in Lugano for the period between January 1, 2024, and March 31, 2024; then, we compared the output of our models to the actual measurements and repeated this procedure for two NABEL stations situated in northern Switzerland (Dübendorf-Empa and Zürich-Kaserne). For these stations, predictions were made for the aforementioned period and the period between January 1, 2023, and December 31, 2023. In most of the cases, the lowest mean absolute errors (MAE) were provided by a non-linear model with 12 components (different powers and linear combinations of NO_2_, NO_X_, SO_2_, non-methane volatile organic compounds, temperature and radiation); the MAE of predicted ozone concentrations in Lugano was as low as 9 μgm^−3^. For the stations in Zürich and Dübendorf, the lowest MAEs were around 11 μgm^−3^ and 13 μgm^−3^, respectively. For the tested periods, the accuracy of the best models was approximately 1 μgm^−3^. Since the aforementioned values are all lower than the standard deviations of the observations we conclude that using ML for complex data analyses can be very helpful and that artificial neural networks do not necessarily outperform simpler models.

## 1 Introduction

Ozone is a major air pollutant, atmospheric oxidant, and anthropogenic (man-made) greenhouse gas that not only threatens human health but also affects agricultural ecosystems (Nuvolone et al., 2018; Yeung et al., 2019; Yan et al., 2022, 2023). Therefore, understanding its complex chemistry is crucial. In this study, we demonstrate the potential of machine learning techniques to predict atmospheric ozone concentrations (target) based on a set of measurement parameters (predictors), thereby enabling a comparison of the models with theory.

Specifically, we describe how machine learning techniques can be applied to identify relevant measurement parameters for forecasting the target variable and to construct different predictive models based on these parameters, including artificial neural networks. Our goal is to present a generalizable approach that can be applied to any dataset with a similar structure. Data analysis was conducted using the programming language *R* (version 4.3.2); for deep learning, the *Python* library *TensorFlow* with its Application Programming Interface *Keras* was used (*TensorFlow* version 2.13.1 and *Python* version 3.9). Our most important *R* scripts are available on GitHub.^1^

Ozone is particularly interesting because atmospheric ozone chemistry involves various substances and reactions. In the stratosphere, ozone is mainly produced by photo-dissociation of molecular oxygen; sunlight with wavelengths λ below 250 nm splits molecular oxygen into elemental oxygen (McConnell and Jin, 2008):


(1) $$\begin{array}{l} {\text{O}}_{2}+\text{hν}\to \text{O}+\text{O},\text{ λ}<250\;\text{nm}. \end{array}$$

In this reaction, hν denotes the photon energy, whereby h stands for the Planck constant and ν = c/λ denotes the frequency. Next, through an additional molecule *M*, each of the resulting oxygen atoms can recombine with an oxygen molecule to form ozone (Solomon, 1999):


(2) $$\begin{array}{l} \text{O}+{\text{O}}_{2}+\text{M}\to {\text{O}}_{3}+\text{M}. \end{array}$$

In the troposphere and the lower stratosphere, O_2_ cannot be photo-dissociated; as a result, ozone formation arises from chemical reactions breaking the O_2_ bond; the main reaction sequence in these regions of the atmosphere is (McConnell and Jin, 2008):


(3) $$\begin{array}{l} \text{CO}+\text{OH}\to \text{C}{\text{O}}_{2}+\text{H}{\text{O}}_{2} \end{array}$$

(4) $$\begin{array}{l} \text{NO}+\text{H}{\text{O}}_{2}\to \text{N}{\text{O}}_{2}+\text{OH} \end{array}$$

(5) $$\begin{array}{l} \text{N}{\text{O}}_{2}+\text{hν}\to \text{NO}+{\text{O}}_{3},\text{ λ}<430\;\text{nm} \end{array}$$

Radicals with a similar structure as HO_2_ originating from oxidized hydrocarbon molecules can undergo similar reactions (McConnell and Jin, 2008). There exist many more reactions that lead to the formation of ozone as well as reactions that lead to its destruction; for instance, in the troposphere the reaction:


(6) $$\begin{array}{l} \text{N}{\text{O}}_{2}+{\text{O}}_{3}\to \text{N}{\text{O}}_{3}+{\text{O}}_{2} \end{array}$$

can take place at night (Finlayson-Pitts and Pitts Jr, 1993). Hence, NO_2_, which is involved in the formation of ozone, also takes part in a reaction that leads to its destruction - this is just one example out of many demonstrating the complexity of atmospheric ozone chemistry. Moreover, the formation and destruction of ozone depends on the abundance of molecules involved in the corresponding reactions. For instance, volatile organic compounds (VOC) oxidize NO to NO_2_. This molecule can, in turn, react with O_2_ to form ozone (Reaction 5) or lead to its destruction (Reaction 6).

Data analysis was performed on data collected by Switzerland's National Air Pollution Monitoring Network (NABEL), which simultaneously measures various greenhouse gases, harmful substances of special interest and atmospheric pollutants in ambient air (Ballaman et al., 2020). The NABEL was established in 1978 to monitor Switzerland's air quality and is operated by the Swiss Federal Office for the Environment as well as by the Swiss Federal Laboratories for Materials Science and Technology (Empa) (Ballaman et al., 2020). Nowadays, this network consists of 16 individual measurement stations distributed all over Switzerland; each of these stations falls into one of the following location types:

- urban location,
- urban location with traffic,
- suburban location,
- rural location with highway,
- rural location situated below 1, 000 m above sea level (ASL),
- rural location situated above 1, 000 m ASL,
- high mountains.

Moreover, the measurement stations represent different geographical regions (Ballaman et al., 2020). For data analysis and model training, we took data recorded at the urban station Lugano-Università in Lugano, TI, Switzerland. For testing, data recorded at the stations Lugano-Università, Zürich-Kaserne (urban location) and Dübendorf-Empa (suburban location) was used; the latter two stations are located in Zürich, ZH, Switzerland and Dübendorf, ZH, Switzerland, respectively.

Each of the evaluated measurement files contains the measurement dates along with the corresponding daily averages of the following measurement parameters:

- Ozone (O_3_) in μgm^−3^
- Nitrogen dioxide (NO_2_) in μgm^−3^
- Sulfur dioxide (SO_2_) in μgm^−3^
- Carbon monoxide (CO) in mgm^−3^
- Particulate matter ≤ 10 μm (PM10) in μgm^−3^
- Particulate matter ≤ 2.5 μm (PM2.5) in μgm^−3^
- Elemental carbon (EC) in PM2.5 (Soot) in μgm^−3^
- Particle number concentration (CPC) in cm^−3^
- Non-methane volatile organic compounds (NMVOC) in ppm
- Nitrogen oxides (NO_X_) in $\text{μg}{\text{m}}^{-3}\text{eq}.\text{N}{\text{O}}_{2}$
- Temperature (TEMP, T) in °C
- Precipitation (PREC) in mm
- Radiation (RAD, I) in Wm^−2^

We indicate concentrations of the aforementioned parameters using square brackets (e.g., [NO_2_]).

Currently, the only station recording all of these parameters is the station Lugano-Università; therefore, models were trained with data from this station. Data sets with the complete set of parameters are available from the year 2016 onwards; the data were downloaded from the website of the Federal Office for the Environment (Federal Office for the Environment, 2024) on April 3, 2024.

**Figure 1:**
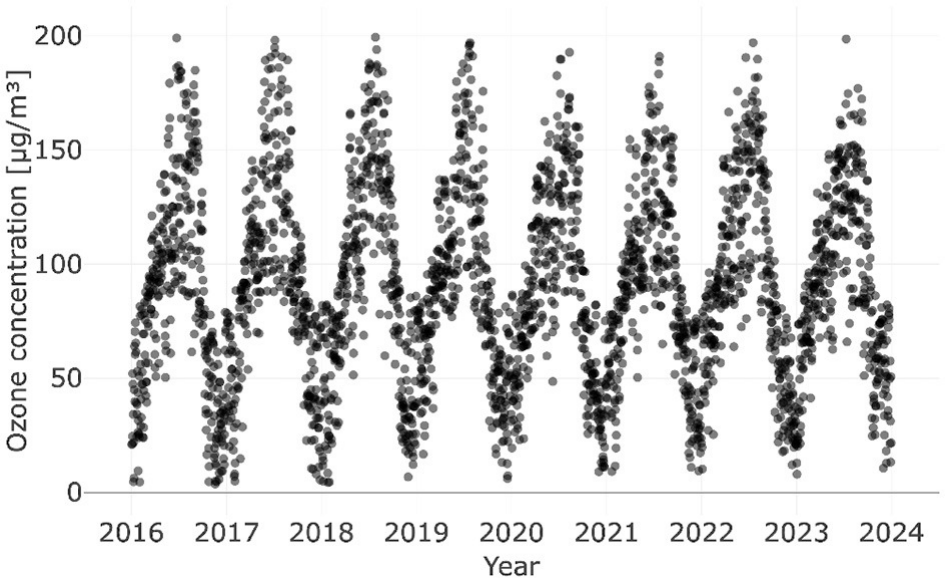
Daily averages of ozone concentrations recorded at the NABEL station in Lugano between January 1, 2016, and December 31, 2023.

When we inspected the data recorded at the aforementioned station, we noticed that with the exception of particulate matter-related parameters (PM10, PM2.5, EC, and CPC) only a few data points are missing (typically less than 1% per year). From the year 2002 onwards, PM10 concentrations are available and the CPC records started in 2005; EC followed in 2011 and PM2.5 in 2016. Between January 1, 2016, and December 31, 2023, data were recorded on 2907 days; on less than 17% of the days, values of certain measurement parameters are not available. Over 60% of these data points correspond to the continuous period between November 3, 2021, and September 8, 2022 – almost a full year – while the remaining data points are rather heterogeneously distributed across various measurement parameters and years. For simplicity, we excluded the corresponding data instead of applying data imputation (2479 entries instead of 2907), as ozone exhibits a distinct yearly cycle (see Figure 1). Although the impact is expected to be minimal, it cannot be ruled out that omitting these data could introduce bias without a more detailed analysis.

As mentioned at the beginning of the introduction, the primary tool used in this work is machine learning (ML), a rapidly growing field that encompasses a wide range of techniques and methods. In recent years, machine learning – particularly deep learning—has gained significant importance in daily life, industry, and research. Deep learning is a subset of machine learning, which is in turn a subset of artificial intelligence (AI) (Chollet, 2021). According to Chollet (2021), AI is a general field that can be described as 'the effort to automate intellectual tasks normally performed by humans'. Generally, machine learning algorithms aim at finding rules in a (large) set of examples by mapping input to target data; these rules can then be applied to data the algorithm has not seen (Chollet, 2021). For this purpose, machine learning algorithms normally need input data, the expected output data (target) as well as a way of measuring the algorithm's performance to adjust the corresponding machine learning algorithm; the latter step is called “learning” (Chollet, 2021). From a mathematical point of view, such algorithms are exposed to many examples to find accurate representations of the input data using a set of transformations (Chollet, 2021). Hence, learning can also be understood as an automatic search process for an optimal data transformation, which produces an output that is as close as possible to the expected values; this search is performed within a given space of possibilities and is guided by the measure of performance (Chollet, 2021).

Machine learning techniques have been developed to address a multitude of problems across various fields. Yet, their adoption in climate science has been relatively slow, despite the potential benefits and the large size of certain data sets (Abbot and Marohasy, 2017). Nevertheless, machine learning has for example been used for climate change analyses (Bochenek and Ustrnul, 2022), for weather prediction (Dueben and Bauer, 2018), and the forecasting of different climatic variables (Abbot and Marohasy, 2017).

A publication resembling our study is that by Mo et al. (2024), who investigated various machine learning and statistical models to identify relevant water quality parameters for predicting the water quality index. Their findings indicate that the most important predictors are turbidity, dissolved oxygen, and nutrients, with the highest predictive accuracy achieved using tree-based ensemble methods, such as random forests and gradient boosting (Mo et al., 2024). Additionally, Mo et al. (2024) demonstrate that prediction accuracy may vary depending on both the grade and the season.

Another instructive and data-driven study is that by Song et al. (2024), who describe their stepwise approach to predicting hourly PM2.5 concentrations. By considering various factors, including cyclical trends, Song et al. (2024) first constructed predictors and applied least squares fitting. From their data, which exhibited outliers and heteroscedasticity, Song et al. (2024) inferred that an adjusted robust heteroscedastic autoregressive spatiotemporal model might be appropriate. This model ultimately proved to fit their data well and was effective for predicting hourly PM2.5 concentrations (Song et al., 2024).

Furthermore, the utility of machine learning techniques in the energy sector has been highlighted in several works, including the study by Sun et al. (2023) on wind speed prediction. Wind speed is known for its non-stationary and irregular fluctuations, which pose challenges for accurate forecasting. Sun et al. (2023) demonstrated that by employing a combined prediction framework, which integrates grid search for hyperparameter selection and a ranking-based adaptive cuckoo search algorithm, predictive performance can be significantly improved.

In the first section of this paper, we outline our methods. Next, we present and discuss the results. Our conclusions are given in the last section of our work.

## 2 Methods

In this section, we outline the methods and models that we used to analyze the data.

### 2.1 Model types

Our models can be divided into three categories:

- *Linear models*. The target variable of a linear model is linear in the predictor variables. Denoting the target as *y*, the *p* predictors as *x*_*i*_ (*i* ∈ [1, *p*]) and the corresponding coefficients as *w*_*i*_, a linear model can be expressed as:
(7) $$\begin{array}{l} y=w_{0}+\overset{p}{\underset{i=1}{\sum }}w_{i}\cdot x_{i}. \end{array}$$
- *Non-linear models*. The predictor variables of non-linear models can be the arguments of non-linear functions (e.g., square root); furthermore, such models may contain interactions between different predictors (e.g. multiplication of two predictors). Usually, non-linear models are still relatively easy to interpret, though.
- *Artificial neural networks*. Nowadays, there are multiple types of artificial neural networks (ANN). Our base model is the multilayer perceptron (MLP), which is composed of successive layers that are used for learning meaningful, layered representations from data in a multi-stage way (see Section 2.2); depending on the number of layers (depth) the learning process is referred to as “deep learning” or “shallow learning” (Chollet, 2021). Although there is no evidence that biological neural networks (BNN) learn in this way (Chollet, 2021), components of ANNs and BNNs are similar to some extent. The receptors, the neural net, the neurons and the effectors of a BNN can be interpreted as the input layer, the processing layer(s), the processing element and the output layer of an MLP, respectively (Guresen and Kayakutlu, 2011). To model the synapses, dendrites, cell body, axon and threshold value of a BNN, the MLP uses the weights, summing function, activation function, output and bias, respectively (Guresen and Kayakutlu, 2011). In principle, linear and non-linear models can be interpreted as shallow neural networks consisting of an input layer, a single hidden layer and an output layer (Biehl, 2023).

### 2.2 Artificial neural networks

As stated previously, an MLP consists of different layers, which in turn contain a certain number of units (nodes); the number of units in the first and the last layer corresponds to the number of predictors and target variables, respectively. Between the input and the output layer, there is at least one hidden layer with a non-specified number of units. A classical MLP is a fully connected feedforward-only (FFO) neural network (NN), which means that information processing occurs in one direction only (Almeida, 2020) and that all nodes of a layer are connected to the nodes of the previous layer. The input of each node corresponds to the sum of the outputs of the previous layer's nodes that are in turn multiplied by individual weights (integers or floating point numbers); furthermore, a bias term is added to this sum (Almeida, 2020). Before passing the result to the next layer, the value is processed by a non-linear activation function (see Section 3.2); one of the most common activation functions in deep learning is the rectified linear unit (relu) (Chollet, 2021). Basically, the weights control the transformations that the NN applies to the data (Chollet, 2021). To determine the weights that provide a minimal discrepancy between the network's output(s) and target(s), the network has to be trained. This training is performed on a certain fraction of the original data set (training set), whereas the remainder is used for the evaluation of the model (test set). At the beginning of the training, random values are assigned to the weights and then an iterative process (training loop) is initiated (Chollet, 2021). Each of these iterations consists of a two-step process (Chollet, 2021): first, a batch of training samples is fed to the network to assess the network's performance for the current weights (forward propagation step). Then, a loss function implementing a distance score (e.g. mean squared error) is used to calculate the deviation of the model's predictions from the targets (loss); for this purpose, the so-called 'validation set' is used, which corresponds to a certain fraction of the training set that was not used for training. The loss is in turn used as a feedback signal for the optimizer, which is the central algorithm of a neural network and determines how the weights should be updated to obtain a lower loss in the next iteration (backward propagation step); this optimizer is normally based on gradient descent (Chollet, 2021). An epoch is defined as an iteration over the entire training data (except for the validation set), which is in turn divided into batches; after each batch, the weights of the network are updated (Chollet, 2021). The minimum batch size is one data point and the maximum size is the entire training set.

It is worth noting that there exist two types of values that influence the network's performance, namely values that are learned during the aforementioned iterative process (parameters) and values that are set by the ANN's designer (hyperparameters) (Chollet, 2021). Examples of hyperparameters are the number of hidden layers, the number of units per layer, the activation functions, the number of epochs and the batch size.

Besides classical MLPs, which process inputs independently and implement an unidirectional flow of information through the network, there also exist more sophisticated networks. For instance, recurrent neural networks (RNN) keep states by using loops (Almeida, 2020). Using *Keras* it is not only possible to build simple MLPs with fully connected (densely connected) layers but also to add more complex layers. We explored the following network types (layers):

- *Convolutional Neural Networks (CNN)*. It is computationally intensive to train ANNs with a large number of nodes; in this case, convolution may help. In a convolutional layer, the number of input values is reduced by employing a filter (kernel) that sequentially traverses the input values and compresses the information through convolution; this filter can be interpreted as a matrix whose entries have to be learnt (Montesinos López et al., 2022). The dimension of the resulting output depends on the size of the filter and on the stride length, which corresponds to the number of steps by which the filter is shifted when the input values are processed (Montesinos López et al., 2022). After convolution, the output can be compressed further through “pooling”; two common options are “maximum pooling” and “average pooling”, which split the values into different subsets and then compute the maxima and averages of these subsets, respectively (Montesinos López et al., 2022). CNNs are often used for image processing (Montesinos López et al., 2022). Thus, to pass the output to a densely connected layer (or to the output layer), the multidimensional output of the convolutional layer is normally converted to a vector; this is known as “flattening” (Montesinos López et al., 2022).
- *Simple Recurrent Neural Network*. Recurrent layers use preceding data for prediction (Boden, 2002). In a simple RNN, the output of a unit (prediction of next input) is not passed to the next layer, but weighted and then added to the next weighted input value (Boden, 2002). In this loop, the weights and biases are shared across every input and the process continues until all available data points are used; only then the result is passed to the next layer (Boden, 2002).
- *Long Short-Term Memory (LSTM)*. The disadvantage of a simple RNN is that the gradient calculated by the optimizer may disappear or explode; the reason for this is the data's long-term dependence on the network (Wang et al., 2022). Whether the gradient increases or decreases depends on the weights' size (Hochreiter and Schmidhuber, 1997). LSTM circumvents this problem through a gating mechanism that controls the deletion and preservation of data; LSTM uses three gates, which are denoted as “input gate”, “forget gate” and “output gate” (Wang et al., 2022).
- *Gated Recurrent Unit (GRU)*. An alternative to LSTM is GRU, which also makes use of a gating mechanism to solve the problem of increasing and decreasing gradients but is less complex than LSTM; it combines the input gate and the forget gate of an LSTM into one gate, which is called “update gate” (Wang et al., 2018). Moreover, due to the simpler structure of GRU, the training time can be reduced (Wang et al., 2022).

For debugging and the generation of boilerplates we sporadically used *Microsoft Copilot*, which is integrated into *Microsoft Edge* and uses *OpenAI's* large language model *GPT-4 (Turbo)*.

Due to overfitting (high variance), excellent performance of the model on the training set does not necessarily imply good performance on the test set (Gareth et al., 2013). Two techniques that may help to select solutions that generalize well are the following (Salehin and Kang, 2023):

- *Regularization*. A penalty term can be added to the loss function that penalizes complex solutions; consequently, simpler models will be favored. There exist different measures of complexity; a common one is the ℓ_2_ complexity that corresponds to the sum of squared weights. The penalty term involves a hyperparameter λ through which the penalization can be controlled.
- *Dropout*. When implementing dropout, during training certain nodes are randomly set to zero; the corresponding values are used during forward and backward propagation. To obtain values that are comparable to those without dropout, the non-zero values have to be scaled. The probability for setting nodes to zero is a hyperparameter.

### 2.3 Model assessment

As already mentioned, models should always be assessed using the test and not the training set; the reason is that models can be created that perfectly fit the training data, which include the measurement errors (Gareth et al., 2013). Common ML techniques for the assessment of models are cross-validation (estimation of test error) and the bootstrap (estimation of parameter accuracy), both of which are resampling methods (Gareth et al., 2013):

- *K-fold cross-validation*. To perform *k*-fold cross-validation (CV), the observations are randomly split into *k*, equally-sized groups (folds) over which one iterates. In each iteration, *k* − 1 groups are used for model fitting and the remaining group for model validation; models are commonly validated using the mean squared error:
(8) $$\begin{array}{l} \text{MSE}=\frac{1}{n}\text{RSS}=\frac{1}{n}\overset{n}{\underset{i=1}{\sum }}{\left(y_{i}-{ŷ}_{i}\right)}^{2}. \end{array}$$
  In Equation (8), RSS denotes the residual sum of squares, *n* the number of observations in the training set, *y*_*i*_ the i-th observation (target value), *x*_*i*_ the i-th predictor value and ŷ_*i*_ the corresponding model prediction. In general, there are several predictors; in this case, *x*_*i*_ is not a scalar but a vector. The *k*-fold CV estimate corresponds to the average of the *k* mean squared errors:
(9) $$\begin{array}{l} \text{CV}=\frac{1}{k}\overset{k}{\underset{j=1}{\sum }}\text{MS}{\text{E}}_{j}. \end{array}$$
  Leave-One-Out Cross-Validation (LOOCV) is a special case of *k*-fold CV, which uses the total number of observations as *k*. Another similar technique is the validation set approach, which randomly splits the original data set into a training and a validation set; the former set is used for model fitting and the latter for model assessment. Using the validation set approach the model is only assessed on a single set.
- *Bootstrap*. The bootstrap is a statistical tool for estimating the uncertainty associated with a statistical learning method or an estimator. The bootstrap is widely applicable and particularly useful if the variability cannot be estimated with standard formulas or if these formulas are difficult to derive. The bootstrap consists in repeatedly sampling *N* observations from the original data set with replacement, where *N* corresponds to the total number of observations; then, the statistic of interest is calculated for all of the bootstrap data sets that were created in this way. As a last step, the uncertainty of this statistic is estimated using a measure of variability like the standard deviation or the standard error. Hence, one of the advantages of the bootstrap is that the estimation can be performed on the original data set without the need for additional data.

### 2.4 Data analysis

In this subsection, we outline the methods that we used for data analysis. Hereafter, we denote the number of potential predictors for ozone concentrations as *p*; for the full data set, *p* is equal to 12 (see Section 1). All explanations of this subsection follow Gareth et al. (2013).

#### 2.4.1 Best subset selection

Greedy best subset selection is an iterative method that performs *p* iterations; it starts with the model containing no predictors and calculates the residual sum of squares (or the R^2^ value); in the next iteration it tests all models containing one predictor, then all models containing two predictors and so on. In each iteration, the model with the smallest RSS (or largest R^2^) is determined. Eventually, the best of the *p* models is selected using training-error-adjusted statistics like the adjusted R^2^ value, Mallow's *C*_*p*_, the Akaike information criterion (AIC) or the Bayesian information criterion (BIC). All of these statistics involve the calculation of the training RSS but use different penalty terms; these terms increase with the number of predictors to adjust for the corresponding reduction in the training set's RSS.

Alternatively, the best model can be selected using the validation set approach or cross-validation. *K*-fold CV begins with randomly assigning data points to one of the k folds and then carries out best subset selection within each of these folds; then, for each model size, the average of the *k* test MSEs is computed. After determining the model size yielding the lowest MSE average, best subset selection is carried out on the full data set to obtain the model of that size. The validation set approach follows a similar procedure.

Please note that we use greedy best subset selection because the number of predictors is low; for data sets with many predictors, stepwise techniques can be used instead, which are computationally more efficient.

#### 2.4.2 Shrinkage methods

**Figure 2:**
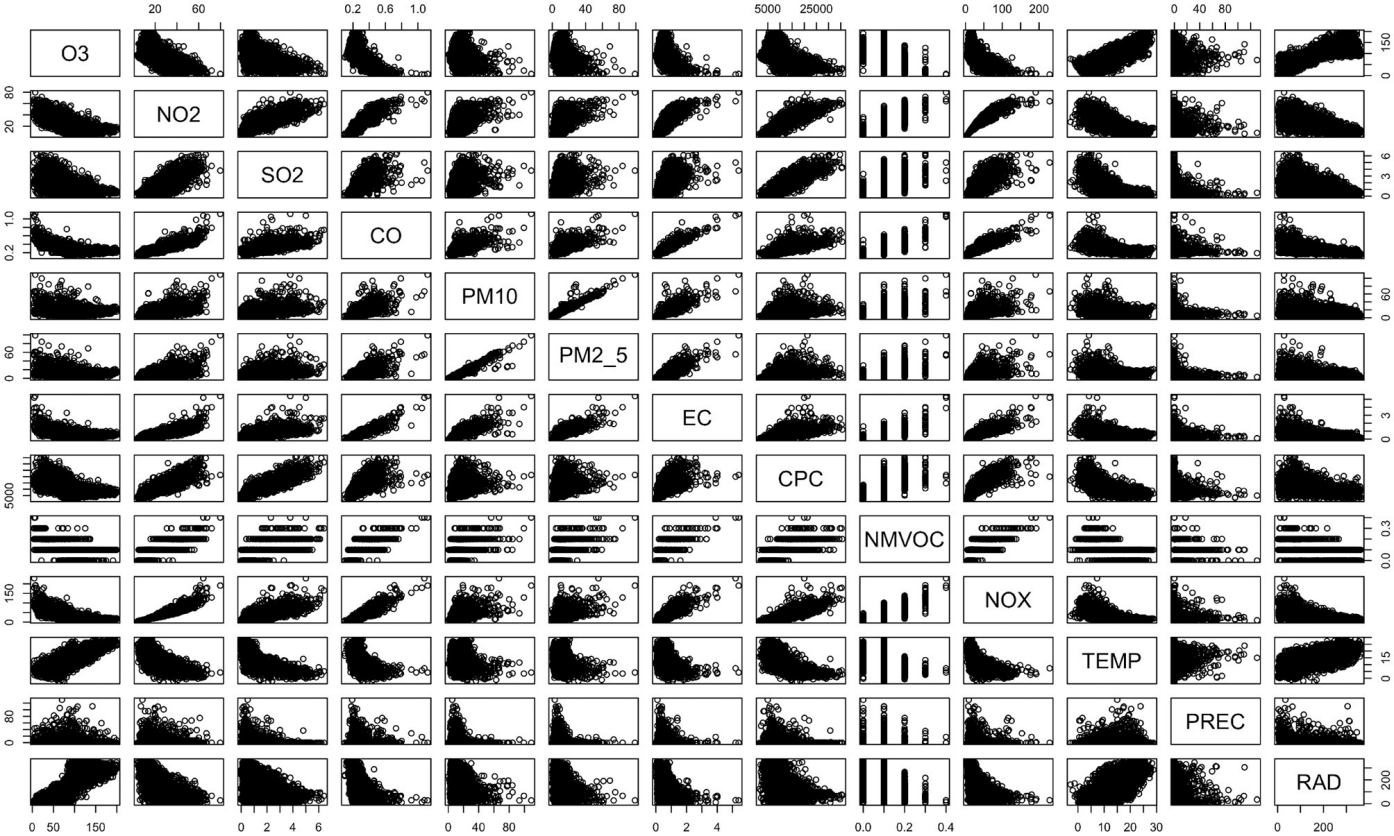
Correlations of daily averages of all measurement parameters recorded at the NABEL station in Lugano between January 1, 2023, and December 31, 2023. The parameters are abbreviated as indicated in Section 1.

If certain features of the data set are not related to the target, shrinkage methods can be used to regularize (constrain) the coefficient estimates. During data inspection, we noticed that not all measurement parameters correlate with ozone (see Figure 2) and therefore assumed that certain features might be irrelevant. For this work we explored two shrinkage methods, namely ridge regression and the lasso; while least squares minimizes the RSS to obtain coefficient estimates, these methods minimize the sum of the RSS and a so-called “shrinkage penalty”. The shrinkage penalty used by ridge regression is the sum of the squared coefficient estimates (without intercept) multiplied by a tuning parameter λ ≥ 0 (ℓ_2_ norm); in contrast, the lasso uses the sum of the absolute values of the coefficients multiplied by λ (ℓ_1_ norm). The larger the tuning parameter, the larger the shrinkage penalty and the larger the resulting RSS; consequently, solutions with small coefficients will be favored. The main difference between ridge regression and the lasso is that only the latter method can be used for variable selection; ridge regression always provides non-zero coefficients for all predictors, whereas the lasso may shrink certain coefficients to zero.

#### 2.4.3 Dimension reduction methods

Principal Component Regression (PCR) is a method that can help to reduce the number of dimensions; this method involves computing principal components (linear combinations of predictors) and creating a new linear regression model (least squares fitting) using these components. Unlike the lasso it is not a feature selection method since the principal components are linear combinations of all predictors; thus, the number of predictors is not reduced. In principle, the first component carries the most information because it points in the direction along which the observations show the largest variation; the second principal component is perpendicular to the first component and tries to capture as much of the remaining information as possible. Given that the number of predictors is larger than two, further principal components can be constructed accordingly. Under the assumption that the directions along which the predictors vary the most are indeed associated with the target variable, a few principal components might be sufficient to construct a model that explains most of the observed variability. A common method to select the number of principal components is cross-validation.

An alternative to PCR is Partial Least Squares (PLS); the main difference is that PLS includes the target variable to supervise the identification of principal components. As a first step, PLS standardizes the predictors and uses simple linear regression to compute the coefficients of the first principal component. Before calculating the second principal component, each variable is adjusted for the first principal component by taking the residuals that are obtained when each variable is regressed on the first principal component; then, the second principal component is calculated in the same way as the first one. To obtain the remaining components this procedure has to be repeated. The minimal number of principal components that should be included in the model can then be determined using cross-validation.

## 3 Results

The data recorded in 2023 suggest that ozone correlates with different measurement parameters like radiation, temperature and nitrous oxides (see Figure 2); the corresponding R^2^ values (coefficients of determination) are 0.72, 0.6 and 0.43, respectively. For the prediction of ozone, other potentially relevant parameters are CO, CPC, EC and SO_2_; these parameters yield R^2^ values between 0.13 and 0.32. Correlations between ozone and the other parameters result in R^2^ values below 0.009 (precipitation, PM2.5 and PM10). Please note that inspecting all correlations in data sets with many more parameters is hardly possible; the larger and more complex the data set, the more difficult to study the data without machine learning or similar approaches.

Due to the observed correlations between ozone and various measurement parameters, the question arises which model describes the relationship best. We used daily averages recorded at the NABEL station in Lugano between January 1, 2016, and December 31, 2023, along with the machine-learning-based methods presented in Section 2 to evaluate different models and to perform variable selection; we only considered days on which all parameters were recorded (approximately 83% of 2907 entries). For the sake of simplicity, in this section, we drop the units of all metrics (e.g. MSE and RSS).

It is worth mentioning that the ozone concentrations recorded on subsequent days are similar (see Figure 1); thus, the error terms might be correlated and the true standard errors could be higher than the estimates provided by linear regression models (Gareth et al., 2013).

In what follows, we first analyze the data to construct linear and non-linear models; then, we focus on artificial neural networks.

### 3.1 Linear and non-linear models

The results of various ML techniques are reported in the first part of this subsection; we subsequently use these results to build different linear and non-linear models for the prediction of atmospheric ozone concentrations.

#### 3.1.1 Best subset selection

As a first step, we used greedy best subset selection to determine the predictors that should be included in a linear model; according to the adjusted R^2^ value, the best model involves all predictors except for EC (adjusted R^2^ = 0.805); however, the model using only radiation and temperature as predictors is within one standard error (SE = 0.01). When assessing the models with *C*_*p*_, the best model turns out to be the one including all predictors except for EC, CPC and PM10 (*C*_*p*_ = 10); the four-variable model using NO_2_, NO_X_, temperature and radiation as predictors is within one standard error (SE = 138), though. According to BIC, the best model does not involve CO, PM2.5, EC and CPC (BIC = -3988); the BIC of the best model is less than one standard error (SE = 103) apart from the BIC of the four-variable model.

Just like *C*_*p*_, 5-fold CV indicates that the nine-predictor model is most appropriate (MSE = 347); within one standard error (SE = 19) there is again the four-predictor model. In contrast, 10-fold CV selects the model with eight predictors; the minimal MSE is 347, the corresponding SE is 19 and the eight-variable model contains all predictors except for PM2.5, EC and CPC.

In general, the different approaches suggest that the best linear models involve eight to nine predictors and that a four-variable model is usually within one SE. The most important predictors seem to be NO_2_, NO_X_, temperature and radiation; apparently, the least important features are CO and particular-matter-related parameters (PM2.5, PM10, EC and CPC). As expected, the best one-variable model includes radiation.

#### 3.1.2 Shrinkage methods

Applying ridge regression to the standardized data set yields λ = 3.5 and an MSE around 358; we obtained these values when testing 1,000 λ values in the range between 1·10^−2^ and 1·10^10^ using 5-fold as well as 10-fold CV (50/50 training-validation split). The features getting the largest coefficient estimates are NMVOC and CO (order of 10) as well as EC, SO_2_ and temperature (order of 1); all of the other coefficient estimates are at least one order of magnitude lower.

When testing the same range of λ values using the lasso and performing 10-fold cross-validation (50/50 training-validation split), one obtains an MSE of roughly 346. The λ value is merely around 0.08 and the lasso only shrinks the PM2.5 coefficient to zero. The largest coefficients are again those associated with NMVOC and CO (order of 10); the coefficients of EC, SO_2_ and temperature are on the order of 1. Similar results are obtained using 5-fold CV.

All in all, the results of ridge regression and the lasso do not agree with the results of best subset selection. Moreover, both methods assign relatively low weights to radiation (usually on the order of 0.1), which shows the strongest correlation with ozone. The mean squared errors of the models obtained with best subset selection (CV) and with the two shrinkage methods are comparable, though. In addition, it is worth noting that the lasso shrinks the coefficient of PM2.5 to zero, even though the coefficient estimates of CPC and precipitation provided by ridge regression are closer to zero.

#### 3.1.3 Dimension reduction methods

Performing PCR with 10-fold CV indicates that 11 principal components should be included in the model (around 81% of variance explained); the test MSE of this model is roughly 344. Calculating the test errors of models with fewer principal components shows that none of the smaller models is within 1 SE. However, we noticed that models with less than five principal components lead to distinctly higher mean squared errors. The five-component model's test MSE is around 383 (78% of variance explained) and including only the first principal component results in a test MSE of around 900 (45% of variance explained); hence, the latter model lacks a considerable amount of information.

According to PLS with 10-fold CV, a nine-component model is suitable; the variance explained and the test MSE are around 81% and 346, respectively. In fact, the 10-component model yields a slightly lower test MSE, namely around 345. The seven-component model is within 1 SE of the 10-component model and yields an MSE of approximately 352 (roughly 81% of variance explained).

In our case, PLS seems to be slightly superior to PCR because similar mean squared errors were attained with fewer principal components; this holds for the training as well as for the test data. We did not pursue dimension reduction methods further since the mean squared errors are not distinctly lower than those provided by the previously discussed methods; in addition, the number of predictors cannot be reduced using these methods.

#### 3.1.4 Model selection

Based on the results of the previous subsections, we built different models for predicting atmospheric ozone concentrations; we also included some basic models to compare their performance to the most complex ones. Whenever the validation set approach was used, 50% of the data were used for validation.

- *Model 1:*

$\left[{\text{O}}_{3}\right]=\left(0.347\cdot I+34.4\right)\text{ μg}{\text{m}}^{-3}$

  The results of Section 3.1.1 suggest that the best simple linear regression model with one predictor includes radiation. Fitting the entire data set yields a slope of $a_{1}=0.347\pm 0.005\text{ μg}{\text{W}}^{-1}{\text{m}}^{-1}$ and an intercept of $a_{0}=34.4\pm 0.9\text{ μg}{\text{m}}^{-3}$; the uncertainties correspond to standard errors. The corresponding p-values of the t-statistic are smaller than 2·10^−6^ and the value of the F-statistic is 5146, which is much larger than 1; these values, as well as the relatively high R^2^ value (0.68), indicate that the predictor is associated with the response. The median of the residuals is −2 μgm^−3^ (first and third quartile values around −17 μgm^−3^ and 15 μgm^−3^, respectively). When compared to the ozone concentration average and its standard deviation (91 ± 42 μgm^−3^), the median of the residuals is small; the residual standard error is considerable, though, namely approximately 24 μgm^−3^. LOOCV, 5-fold and 10-fold CV show that the MSE of the model is roughly 575. We obtained an MSE of around 563 using the validation set approach.
- *Model 2:*

$\left[{\text{O}}_{3}\right]=\left(-210\cdot {\text{I}}^{3}-203\cdot {\text{I}}^{2}+1,720\cdot \text{I}+90.6\right)\text{ μg}{\text{m}}^{-3}$

  Plotting ozone against radiation reveals that a linear function might be an oversimplification of their actual relationship. Thus, we tested a fourth-order polynomial fit; the p-values of all coefficients up to the third order turned out to be lower than 2·10^−6^. Using 10-fold CV for the second-, third- and fourth-order polynomials showed that the mean squared errors are around 559, 542 and 541, respectively. Similarly, the validation set approach provided mean squared errors of roughly 549, 530 and 529, respectively. From this we concluded that the third- and fourth-order polynomials provide similar results and that both models are superior to Model 1; for simplicity, we opted for the third-order model (see first line). For this model, the standard error of the intercept is 0.5 and roughly 23 for the other coefficients. When comparing Model 1 to the third-order polynomial model using a hypothesis test (null hypothesis: no model is superior), the corresponding F-statistic turns out to be around 79 and the associated p-value is below 2.2·10^−6^. The F-statistic is not very large but the p-value is almost zero; hence, the hypothesis test implies that the higher-order model might be superior. This finding is consistent with the outcomes of CV and the validation set approach.
- *Model 3:*

$\left[{\text{O}}_{3}\right]=\left(1.4\cdot \left[\text{N}{\text{O}}_{2}\right]+3.4\cdot \left[\text{S}{\text{O}}_{2}\right]+46\cdot \left[\text{NMVOC}\right]-0.97\cdot \left[\text{N}{\text{O}}_{\text{X}}\right]+2.7\cdot \text{T}+0.202\cdot \text{I}+11\right)\text{ μg}{\text{m}}^{-3}$

  In general, greedy best subset selection implies that the best models do not involve particulate-matter-related parameters (PM2.5, PM10, EC, and CPC); thus, we tested a linear model excluding them. Fitting this model to the data shows that the highest p-values are those associated with CO and precipitation. Computing the mean squared errors using 10-fold CV and the validation set approach results in values around 349 and 343, respectively; when CO and precipitation are excluded from the model the latter value increases to 344. Using NO_2_, NO_X_, temperature and radiation as predictors and calculating the MSE with the validation set approach yields roughly 353; hence, the mean squared error of this model is notably higher than the mean squared errors of the former two models. Comparing the models using 10-fold CV leads to the same conclusions. Adding precipitation and CO to the model did not substantially improve the results. Therefore, we dropped these parameters and only used NO_2_, NO_X_, temperature, radiation, SO_2_ and NMVOC as predictors; the standard errors of their coefficients are 0.1, 0.6, 10, 0.04, 0.1, and 0.006, respectively. The bootstrap (1,000 samples) provided similar values, namely 0.13, 0.5, 10, 0.07, 0.1, and 0.006, respectively. Since NO_2_ and NO_X_ are correlated, we also tested models that only include one of these variables; the same was done for temperature and radiation. However, the validation set approach showed that the mean squared errors of all of these models are comparatively high and lie in the range between 370 and 496.
- *Model 4:*

$\left[{\text{O}}_{3}\right]=\left(-1.8\cdot 10^{-4}\cdot {\left[\text{N}{\text{O}}_{2}\right]}^{3}-1.41\cdot \left[\text{N}{\text{O}}_{\text{X}}\right]+1.8\cdot 10^{-2}\cdot {\left[\text{N}{\text{O}}_{\text{X}}\right]}^{2}-4.4\cdot 10^{-5}\cdot {\left[\text{N}{\text{O}}_{\text{X}}\right]}^{3}+3.0\cdot 10^{-3}\cdot {\text{T}}^{3}+1.4\cdot 10^{-1}\cdot \left[\text{N}{\text{O}}_{2}\right]\cdot \text{T}+1.7\cdot 10^{-2}\cdot \left[\text{N}{\text{O}}_{2}\right]\cdot \text{I}-22\cdot \left[\text{S}{\text{O}}_{2}\right]\cdot \left[\text{NMVOC}\right]+6.9\cdot 10^{-1}\cdot \left[\text{S}{\text{O}}_{2}\right]\cdot \text{T}+3.7\cdot 10^{-1}\cdot \left[\text{NMVOC}\right]\cdot \text{I}-1.16\cdot 10^{-1}\cdot \left[\text{N}{\text{O}}_{\text{X}}\right]\cdot \text{T}-7.3\cdot 10^{-3}\cdot \left[\text{N}{\text{O}}_{\text{X}}\right]\cdot \text{I}+71\right)\text{ μg}{\text{m}}^{-3}$

  Based on Model 3, we tested a model that involves all of its predictors but added all terms up to third order; furthermore, we allowed for interactions between these predictors by adding all possible products of first-order terms. Greedy best subset selection along with *C*_*p*_ shows that the best model contains 21 terms (NO_2_, $\text{N}{\text{O}}_{2}^{2}$, all orders of NO_X_, SO_2_, NMVOC, T, T^2^, I^2^, I^3^, all interactions between temperature and the other predictors, all interactions between radiation and the other predictors as well as the interaction between NO_X_ and NMVOC); it is striking that most interaction terms either involve temperature or radiation. From a chemical perspective, this seems reasonable because temperature and radiation control certain reactions. According to best subset selection, the model with the lowest number of components deviating by less than one SE (roughly 114) from the 21-component model is the 12-component model; using 10-fold CV shows that the mean squared errors of the two models are around 233 and 238, respectively. The validation set approach yields an MSE of around 228 for the more complex model and 234 for the simpler one. Due to similar performance with almost half the number of terms, we selected the 12-component model; fitting this model to the data recorded between January 1, 2016, and December 31, 2023, in Lugano provides the coefficients shown at the beginning of the paragraph. Just as for Models 1 to 3, the coefficients were rounded according to the standard errors of the corresponding fit estimates.

### 3.2 Neural networks

Besides linear and non-linear models we also trained different types of artificial neural networks, which are listed in Table 1; as indicated in Section 2.2, the networks were built using *Keras'* sequential models, which have a layered structure. Hereafter, we denote a network with at least one recurrent layer as RNN and networks with at least one convolutional layer as CNN. As stated at the beginning of this chapter, the data were recorded at the University of Lugano between January 1, 2016, and December 31, 2023; 80% of these data were used for training and the remaining 20% for testing. The test error was assessed using the MSE and during training, 10% of the training set was used for validation.

**Table 1:** Mean squared errors (MSE) of various neural networks for the prediction of ozone concentrations.

**Model**	**Model type**	**Predictors**	**MSE [μg^2^m^−6^]**
5	Feedforward-only neural network	12	167
6	Feedforward-only neural network	12	168
7	Feedforward-only neural network	6	223
8	Recurrent neural network	12	173
9	Recurrent neural network	12	174
10	Recurrent neural network	6	226
11	Convolutional neural network	12	190
12	Convolutional neural network	12	192

The networks were trained with data recorded at the NABEL station in Lugano from January 1, 2016, to December 31, 2023; the MSEs were obtained with the set of hyperparameters that performed best on the training set and were computed using 20% of the data, which were not used during training.

For each neural network type, we evaluated several models, which were obtained by varying one hyperparameter at a time (number of epochs, batch size, units per layer, activation function, optimizer and regularization method). The activation functions we tested are:

- *Hyperbolic tangent*: $\text{tanh}\left(x\right)=\frac{e^{x}-e^{-x}}{e^{x}+e^{-x}}$
- *Sigmoid:* $\text{sigmoid}\left(x\right)=\frac{1}{1+e^{-x}}$
- *Rectified linear unit*: relu(*x*) = max(0, *x*)
- *Leaky rectified linear unit*: Defining α as a small positive constant, the leaky rectified linear unit is given by
(10) $$\begin{array}{l} lrelu\left(x\right)=\{\begin{array}{cc} x, & \text{if}x>0\\ \alpha \cdot x, & \text{otherwise} \end{array} \end{array}$$
- *Swish*: swish(*x*) = *x*·sigmoid(α·*x*), where α is a constant
- *Mish*: mish(*x*) = *x*·tanh(ln(1 + *e*^*x*^))
- *Softmax*: $\text{softmax}\left(x_{i}\right)=e^{x_{i}}\cdot {\left(\overset{N}{\underset{j=1}{\sum }}e^{x_{j}}\right)}^{-1}$, where *x* is an *N*-dimensional vector and *i* ∈ [1, *N*]
- *Exponential linear unit*: Defining α as a positive constant, the exponential linear unit is given by
(11) $$\begin{array}{l} elu\left(x\right)=\{\begin{array}{cc} x, & \text{if}x>0\\ \alpha \cdot \left(e^{x}-1\right), & \text{otherwise} \end{array} \end{array}$$

The optimizers we used for our studies are

- *RMSprop*. The RMSprop (rms) optimizer uses plain momentum instead of Nesterov momentum and divides the gradient by the root of the moving average of the square of gradients (Keras., 2024d).
- *Adam*. This optimizer is based on a stochastic gradient descent method that uses adaptive estimation of moments up to second order. In principle, the adam optimizer is RMSprop with momentum (Keras., 2024b) (Keras., 2024c).
- *Nadam*. Basically, the Nadam optimizer is Adam with Nesterov momentum (Keras., 2024c).
- *Adadelta*. The Adadelta optimizer is also based on stochastic gradient descent and uses an adaptive learning rate per dimension (Keras., 2024a).

Regarding RNNs, we tested simple RNN, LSTM and GRU layers. For each NN type (FFO NN, RNN and CNN), we selected the set of hyperparameters that provided the lowest MSE during our tests. Actually, selecting models based on single runs should be avoided because it is not possible to test all possible models and combinations of hyperparameters; in addition, if the same model is trained multiple times, different weights are obtained, which eventually results in different test errors. To estimate the variability of the test error, we trained the three selected models 10 times with a fixed set of hyperparameters; for each model type, we saved the weights of different models that yielded low mean squared errors (see models in Table 1). From the training repetitions, we learned that the standard deviations of the models' mean squared errors are in the range of 7 to 15. The best FFO NN, RNN and CNN provided test MSEs around 167, 173, and 190, respectively; we denote the corresponding models as Model 5, Model 8 and Model 11, respectively (see Table 1).

Since Lugano is the only station at which all 12 measurement parameters are recorded, we also trained FFO NNs and RNNs with six instead of 12 predictors (inputs), namely those that are part of Model 3 (NO_2_, NO_X_, SO_2_, NMVOC, temperature and radiation). The best FFO NNs and RNNs (Model 7 and Model 10), yielded mean squared errors around 223 ± 4 and 226 ± 10, respectively; the uncertainties correspond to the standard deviations of the 10 training repetitions.

### 3.3 Evaluation of model performance

To compare our models and to evaluate their performance, we predicted daily averages of ozone concentrations for the period between January 1, 2024, and March 31, 2024, using data recorded at the NABEL station in Lugano; the corresponding test errors are shown in Table 2.

**Table 2:** Averages, mean squared errors (MSE), and mean absolute errors (MAE) of ozone concentrations predicted by different linear and non-linear models as well as by various artificial neural networks (NN); the models were numbered as in the main text and were trained using data recorded between January 1, 2016, and December 31, 2023, at the NABEL station in Lugano (2,479 records).

**Model**	**Type**	**Predictors/components**	**MSE [μg^2^m^−6^]**	**MAE [μgm^−3^]**	**Average [μgm^−3^]**
1	Linear	1	342	15	66
2	Non-linear	3	367	16	66
3	Linear	6	202	11	58
4	Non-linear	12	144	9	61
5	Feedforward-only NN	12	150	10	66
6	Feedforward-only NN	12	145	10	67
7	Feedforward-only NN	6	455	18	62
8	Recurrent NN	12	182	11	65
9	Recurrent NN	12	163	9	63
10	Recurrent NN	6	7802	78	139
11	Convolutional NN	12	167	11	61
12	Convolutional NN	12	182	11	64

All displayed values were calculated on the test set recorded between January 1, 2024, and March 31, 2024, at the same station; the average of the observed ozone concentrations during this period is 65 ± 21 μgm^−3^ (1σ). In the third column, we listed the number of predictors (inputs) of NNs and the number of components (excluding the intercept) of linear as well as non-linear models.

To assess our models on data gathered at locations other than Lugano, we used records of the NABEL stations Zürich-Kaserne and Dübendorf-Empa located in northern Switzerland; we selected these two stations because they record all measurement parameters except for CPC. In Table 3, we present performance tests that were carried out for the period between January 1, 2023, and December 31, 2023, as well as between January 1, 2024, and March 31, 2024; regarding neural networks, only those with six predictors are reported because Lugano is the only station at which all 12 predictors are recorded.

**Table 3:** Averages (AV), mean squared errors (MSE), and mean absolute errors (MAE) of ozone concentrations predicted by different linear and non-linear models as well as by various artificial neural networks (NN); the models were numbered as in the main text and were trained using data recorded between January 1, 2016, and December 31, 2023, at the NABEL station in Lugano (2,479 records).

**Model**	**Year**	**Z, MSE [μg^2^m^−6^]**	**Z, MAE [μgm^−3^]**	**Z, AV [μgm^−3^]**	**D, MSE [μg^2^m^−6^]**	**D, MAE [μgm^−3^]**	**D, AV [μgm^−3^]**
1	2024	442	18	62	508	18	57
2	2024	492	19	61	534	19	55
3	2024	465	18	53	373	16	52
4	2024	188	11	63	300	13	60
7	2024	243	12	70	314	14	64
10	2024	239	12	68	353	15	69
1	2023	446	17	89	510	20	55
2	2023	492	18	89	544	20	54
3	2023	456	18	87	454	18	49
4	2023	329	14	93	324	14	58
7	2023	342	14	96	346	14	61
10	2023	346	14	95	303	13	63

All displayed values were calculated on test sets recorded at the NABEL stations Zürich-Kaserne (Z) and Dübendorf-Empa (D) between January 1, 2023, and December 31, 2023, as well as between January 1, 2024, and March 31, 2024. The averages of the observed ozone concentrations in Zürich in 2023 and 2024 are 88 ± 29 μgm^−3^ and 65 ± 20 μgm^−3^, respectively; the averages recorded at the station in Dübendorf are 63 ± 21 μgm^−3^ and 60 ± 19 μgm^−3^, respectively. The uncertainties of the measured averages correspond to 1σ.

### 3.4 Uncertainty of weight and biases of neural networks

To the best of our knowledge, at the time of writing, *Keras* did not provide estimates for the precision of a neural network's weights and biases. One approach to obtain such estimates is through the bootstrap method.

To test this procedure and obtain a sense of the uncertainty in the weights and biases of our multilayer perceptrons, we trained such a model 10 times using data gathered at the NABEL station in Lugano from January 1, 2016, to December 31, 2023. We then computed the averages and standard deviations of the weights and biases from the 10 runs. Since a network with 32 units in the first layer and 64 units in the subsequent layers generally provided the best results (see Sect. 4), we adopted this configuration for this study. We also used 80 epochs and a batch size of 8. Since such a network contains 10913 weights and biases (including output layer with a single node), in Table 4, we summarize the results by layer rather than listing estimates for individual weights and biases.

**Table 4:** Values (Val.) and standard deviations (Sd.) of weights and biases of a multilayer perceptron with five layers (including the output layer) determined from 10 bootstrap estimates.

**Parameter**	**Count**	**Val. mean, median**	**Val. min, max**	**Sd. mean, median**	**Sd. min, max**
Weights 1st layer	384	0.02, 0.05	-1.20, 1.31	0.13, 0.10	0.02, 0.49
Biases 1st layer	32	0.11, 0.09	-0.44, 0.65	0.078, 0.059	0.008, 0.226
Weights 2nd layer	2,048	-0.02, -0.03	-1.36, 1.44	0.13, 0.11	0.02, 0.63
Biases 2nd layer	64	-0.04, -0.05	-0.69, 0.78	0.11, 0.08	0.02, 0.35
Weights 3rd layer	4,096	-0.01, -0.01	-2.03, 1.44	0.116, 0.088	0.003, 0.802
Biases 3rd layer	64	0.03, 0.04	-0.3, 0.3	0.06, 0.05	0.01, 0.16
Weights 4th layer	4,096	0.01, 0.01	-1.02, 1.17	0.071, 0.054	0.007, 0.565
Biases 4th layer	64	0.03, 0.08	-0.44, 0.38	0.08, 0.08	0.02, 0.17
Weights 5th layer	64	-0.4, 0.1	-2.0, 0.9	0.22, 0.13	0.01, 0.87
Biases 5th layer	1	0.4, 0.4	0.4, 0.4	0.08, 0.08	0.08, 0.08

The network employed the swish, tanh, relu, and mish activation functions, with 32, 64, 64, and 64 units in the corresponding layers, respectively. Training was conducted using data collected at the NABEL station in Lugano from January 1, 2016, to December 31, 2023. For each of the 10 runs, the training utilized 80 epochs and a batch size of 8. The second column shows the number of weights (or biases) for each corresponding layer.

## 4 Discussion

In this section, we first address the interpretability of our models in the context of atmospheric ozone chemistry, followed by a discussion of the ozone concentration predictions for Lugano and two stations in northern Switzerland based on our models. In the last subsections, we provide further insights into neural networks and non-linear models.

### 4.1 Atmospheric ozone chemistry

From a chemical perspective, our models presented in Section 3.3 emphasize the crucial role of radiation in ozone formation, as shown in Reactions 1, 5, both of which are photo-dissociation processes. As expected, temperature is also typically included in the models, since the Earth's atmosphere is heated by the sun, leading to a correlation between radiation and temperature. Furthermore, Reactions 5, 6 indicate that incorporating NO_2_ and NO_X_ into the models is reasonable. Moreover, when nitrogen oxide concentrations are sufficient, NMVOCs serve as important ozone precursors (Zhang et al., 2024). This could be reflected in the high coefficient of NMVOCs in Model 3. In the same model, the coefficient for SO_2_ is also notably high. Since SO_2_ absorbs light in the wavelength range of 180 nm to 390 nm, it influences the aforementioned photochemical reactions and leads to ozone depletion (Huff, 1996). Although it is beyond the scope of this work, further investigation is required to understand why the models assign such significance to NMVOCs and SO_2_, as both indirectly affect ozone concentrations.

According to Reaction 3, carbon monoxide might be relevant for ozone prediction, but it was generally excluded from the models. However, CO is part of a reaction chain that ultimately produces NO_2_, which is commonly included in the models and is eventually photo-dissociated to form ozone. We assume that the information provided by CO is already captured by NO_2_. Similarly, particulate matter parameters were excluded from the models, even though PM2.5 can influence ozone concentrations through heterogeneous reactions and aerosol-photolysis feedback mechanisms (Qu et al., 2023). We believe, however, that the effect of PM2.5 on ozone is too small to be reflected in the models.

### 4.2 Lugano

The results shown in Table 2 suggest that the most precise models for predicting ozone concentrations in Lugano are the non-linear model with 12 components (Model 4) and the RNN with 12 predictors (Model 9); the former model yields the lowest MSE and MAE, which are approximately 144 μg^2^m^−6^ and 9 μgm^−3^, respectively. Between January 1, 2024, and March 31, 2024, an average ozone concentration of 65 μgm^−3^ was recorded; the standard deviation and standard error are roughly 21 μgm^−3^ and 2 μgm^−3^, respectively. Since ozone concentrations evolve (see Figure 1), we also calculated the standard deviation after applying a trend correction to the data and obtained approximately 15 μgm^−3^; the MAE of the best model is approximately 60% of this value. In contrast, the mean absolute errors of the two models with only one predictor (Model 1 and Model 2) are roughly equal to the standard deviation of the trend-corrected ozone observations. For the trend correction, we used a second-order polynomial fit and defined the ozone concentration recorded on the first day as the point of reference.

Furthermore, it is striking that both feedforward-only neural networks with 12 predictors provide similar mean squared errors as the non-linear model with 12 components (Model 4), namely 145 μg^2^m^−6^ (Model 6) and 150 μg^2^m^−6^ (Model 5). In contrast, the more complex CNN models performed worse than the FFO NNs but better than various linear and non-linear models. Model 9 (RNN with 12 predictors) also yielded an MAE around 9 μgm^−3^, but a slightly higher MSE than the best non-linear model (Model 4), namely 163 μg^2^m^−6^ instead of 144 μg^2^m^−6^. Although recurrent neural networks are popular for the analysis of time-series (Wang et al., 2022), predictions of ozone concentrations in Lugano made with Model 10 (RNN with six predictors) are not reasonable (test MSE around 7, 802 μg^2^m^−6^). In general, neural networks with six predictors performed worse than those with 12 predictors, which might be an indication that the former models lack information; this hypothesis cannot be verified, though, since it is not feasible to test all neural networks with six and 12 predictors.

The ozone concentration average predicted by all of the models is close to the measured value except for Model 10. The averages provided by Models 1, 2, 5, and 8 yielded averages that closely matched the observations, up to approximately 1 μgm^−3^. The best result was achieved by Model 8 (RNN model with 12 predictors), which simultaneously yielded a low MAE. The average concentration predicted by Model 4 (non-linear model with 12 components), which turned out to be the most precise model, deviates by roughly 4 μgm^−3^ from the measured average.

### 4.3 Zürich and Dübendorf

Just as in the case of Lugano, the model that yields the lowest MSE and MAE for ozone concentration predictions in Zürich and Dübendorf is generally Model 4 (non-linear model with 12 components). The only exception is the data recorded in 2023 in Dübendorf, in which case the RNN model with six predictors (Model 10) performed best. This is surprising because the test error of this model is very high for the predictions related to Lugano in 2024, although the model was trained with data from this station. In addition, there is a tendency toward higher test errors when compared to the data recorded in Lugano. From this we conclude that our models might incorporate regional information; if so, they are only meaningful for local predictions. However, verifying this hypothesis would require to extend our study to further NABEL stations, which is outside of the scope of this work. Moreover, it might be worthwhile to assess the models' predictions for extended periods in the future.

For the test data recorded in 2024 at the stations in Zürich and Dübendorf, the best models provided an MAE of 11 μgm^−3^ (Model 4) and 13 μgm^−3^ (Model 4), respectively; for the year 2023 the corresponding mean absolute errors are 14 μgm^−3^ (Model 4) and 13 μgm^−3^ (Model 10), respectively. The ozone concentrations recorded in Zürich in 2023 and 2024 were 88 ± 29 μgm^−3^ and 65 ± 20 μgm^−3^, respectively; during the same periods, the ozone concentrations in Dübendorf were 63 ± 21 μgm^−3^ and 60 ± 19 μgm^−3^, respectively. After applying a trend correction to the ozone concentrations recorded in Zürich and Dübendorf during the first three months of 2024, we obtained standard deviations around 16 μgm^−3^ and 17 μgm^−3^, respectively. Hence, the mean absolute errors of the best models remain lower than the corresponding standard deviations, although the differences are slightly smaller than those observed for the data collected in Lugano.

Based on the data recorded in 2024 at the stations in Zürich and Dübendorf, the most accurate model is Model 4; the deviations of the predicted averages from the measured values are in the range between 0 μgm^−3^ and 2 μgm^−3^. In contrast, for the data gathered in 2023, Models 1 to 3 provided the best results for Zürich and Model 10 the best result for Dübendorf; the discrepancies between the average concentrations predicted by these models and the measured values are around 0 μgm^−3^ to 1 μgm^−3^.

In summary, our results suggest that Model 4 (non-linear model with 12 components) and Model 10 (RNN with six predictors) are probably the most appropriate models for predicting ozone concentrations in Zürich and Dübendorf.

### 4.4 Parameter uncertainty of neural networks

The standard deviations of the weights and biases in our multilayer perceptrons are assumed to be less than 10% of the corresponding averages. This assumption is based on bootstrap estimates, which are summarized in Table 4. Additionally, the table also reveals that the largest uncertainties were observed in the weights of the second and third layers, with the ratios of standard deviation to mean values just under 9%. Additionally, by comparing the differences between the means and medians to the range between the corresponding minima and maxima, we infer that the values and standard deviations of the weights and biases are relatively homogeneous. The largest discrepancies occurred in the weights of the fifth layer, where the differences were around 17% for the values and 11% for the standard deviations. However, the values appear to be more uniformly distributed than their standard deviations. On average, the aforementioned metric is around 3% for the values and approximately 5% for the standard deviation when averaged across all layers and parameters.

### 4.5 Comparison of neural networks to non-linear models

As mentioned in Section 3.4, the large number of nodes in a neural network makes it difficult to analyze and interpret such models. Thus, to investigate why certain non-linear models outperformed our complex neural networks, we compared the residuals of predictions from different models made for Lugano between January 1, 2024, and October 31, 2024, rather than basing the analysis on the model parameters.

**Figure 3:**
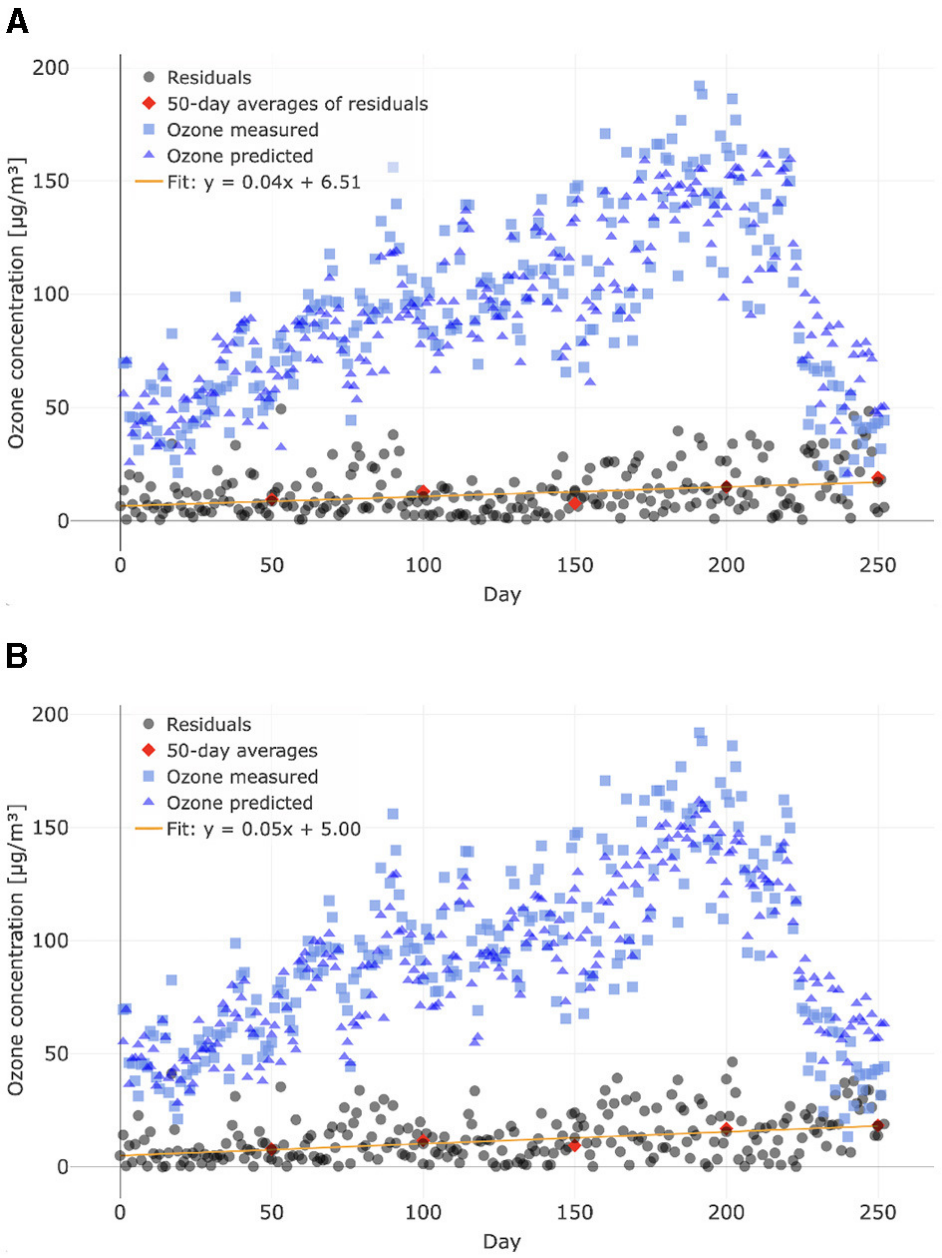
Ozone concentrations and residuals (absolute differences between predictions and targets). Predictions were made for the period between January 1, 2024, and October 31, 2024 for the NABEL station in Lugano using **(A)** Model 8 (recurrent neural network) and **(B)** Model 4 (non-linear model with 12 predictors). For model training, data recorded between January 1, 2016, and December 31, 2023, at the same station were used. The 50-day averages of the residuals were calculated using the 50 preceding the time at which they are plotted.

From Figure 3, which contrasts Model 4 (non-linear model with 12 predictors) with Model 8 (recurrent neural network), it can be observed that the residuals of both models increase with time. In particular, the slopes of the linear fits to the residuals are nearly identical, suggesting that the deterioration in both models is comparable. Although both models capture the significant decrease in ozone in the second half of the year, they apparently fail to fully account for it. Similar observations were made for most of our models. However, when examining the behavior of the two models depicted in Figure 3, it can be seen that the offsets of the residuals are noticeably different. From this, we conclude that the main difference in the performance of the two models arises from a general issue, rather than from a problem concerning a specific period of the year.

## 5 Conclusions

A comparison of the analyses reported in Section 3 with the fundamentals of ozone chemistry outlined in the introduction shows that the machine learning methods generally selected prominent parameters involved in ozone chemistry, such as radiation, temperature, NO_2_, and NO_X_. However, since most of the parameters recorded by the NABEL are relevant to atmospheric ozone chemistry, further work is needed to validate the significance of the selected parameters and their coefficients, assess the meaningfulness of the models and understand the insights they can provide. We suggest particularly focusing on parameters that indirectly affect ozone, such as NMVOCs, SO_2_, CO and particulate matter, whose contributions to ozone formation are less straightforward compared to those of the key parameters directly involved in ozone photochemistry.

Although two RNNs provided low mean absolute errors and led to accurate predictions for ozone concentrations in Lugano, in general, the neural networks did not outperform the best non-linear models. Moreover, the latter models are easily interpretable, which is usually not the case for neural networks with a large number of nodes. Further drawbacks of neural networks are that training is time-consuming as well as computationally intensive; moreover, testing all combinations of hyperparameters is hardly possible and it is difficult to determine whether the performance of the models is close to optimum or not. While we manually tested different model types and hyperparameters, other groups use software packages that automate this task to a certain extent (Abbot and Marohasy, 2017).

For constructing linear and non-linear models, best subset selection turned out to be very helpful because it seemed to successfully drop the least important variables; the more features a data set has, the more difficult it gets to manually construct adequate models.

For the test periods, all of our neural networks and non-linear models with 12 components (Models 4, 5, 6, 8, 9, 11, and 12) yielded ozone concentrations whose mean absolute errors are lower than the standard deviations of the observations; while the standard deviation of ozone concentrations recorded in Lugano between January 1, 2024, and March 31, 2024, is around 15 μgm^−3^ (trend-corrected), the mean absolute errors of the aforementioned models are in the range between 9 μgm^−3^ and 11 μg^2^m^−6^. This statement also holds for data gathered at the NABEL stations in Dübendorf and Zürich between January 1, 2024, and March 31, 2024, as well as between January 1, 2016, and December 31, 2023; when compared to Lugano, the mean absolute errors are typically higher, though. For Zürich and Dübendorf the mean absolute errors calculated on the test sets lie in the range between 11 μgm^−3^ and 14 μgm^−3^. Regarding accuracy, several models predicted concentrations whose averages are within 1 μgm^−3^ of the measured values; the most precise models are not always the most accurate, though.

Since the errors for the northern stations appear to be higher than those for Lugano, whose data were used for training, our models may incorporate regional information. While our work provides a basic approach for machine-learning-based prediction of atmospheric ozone concentrations, it would benefit from a more detailed study to identify the regions for which the models are most suitable. Additionally, it is important to determine how the models should be adjusted to make accurate predictions across all regions and for any time of the year, as predictions were generally more effective for the first half. Since most of the NABEL stations only record a subset of the parameters available at Lugano, expanding our approach to all stations, followed by a comparison of the models, may not be reasonable, as some relevant predictors would be unavailable. A potentially useful technique in this context is transfer learning, which allows a neural network to be adapted to a slightly different problem (Memmert, 2024). Often, most of the network's layers are left unchanged, while a few are re-trained (Weiss et al., 2016). However, in our case, the input layer would have to be adapted as well, since the number of inputs is not the same for all the stations.

Furthermore, it should be assessed whether omitting rows with incomplete data introduces bias, and whether more sophisticated data imputation methods could yield better results. This is a delicate issue, as both missing data and data imputation can result in biases (White et al., 2010; Cummings, 2013). To investigate whether excluding data had such an effect, we recommend omitting a comparable amount of data at different points in the dataset and evaluating whether the results show notable changes.

Although it was not the goal of this study, a comparison of our models with future ozone concentration predictions that incorporate measured ozone concentrations as predictors could be valuable, as atmospheric ozone exhibits a distinct yearly cycle (see Figure 1). For this purpose, recurrent neural networks, commonly used for time-series analysis (Wang et al., 2022), could be particularly useful.

Finally, it would be worthwhile to explore the approach proposed by Dong et al. (2024), who demonstrated that the forecasting accuracy of dissolved oxygen levels could be improved by combining eight artificial neural networks and one statistical model, using Yeung's double-slit experiment optimizer to determine the weights of the individual models.

## Data Availability

Publicly available datasets were analyzed in this study. This data can be found here: https://www.bafu.admin.ch/bafu/de/home/themen/luft/zustand/daten/datenabfrage-nabel.html.

## Footnotes

^1^ https://github.com/stephanunibe/ozone-machine-learning.git
